# Gain-of-function mutations in the ALS8 causative gene VAPB have detrimental effects on neurons and muscles

**DOI:** 10.1242/bio.20137070

**Published:** 2013-12-10

**Authors:** Mario Sanhueza, Luigi Zechini, Trudy Gillespie, Giuseppa Pennetta

**Affiliations:** Centre for Integrative Physiology, Euan MacDonald Centre for Motor Neuron Disease Research, School of Biomedical Sciences, University of Edinburgh, Edinburgh EH8 9XD, UK

**Keywords:** ALS, *Drosophila*, Genetics, Neurodegeneration, VAPB

## Abstract

Amyotrophic Lateral Sclerosis (ALS) is a motor neuron degenerative disease characterized by a progressive, and ultimately fatal, muscle paralysis. The human VAMP-Associated Protein B (*hVAPB*) is the causative gene of ALS type 8. Previous studies have shown that a loss-of-function mechanism is responsible for VAPB-induced ALS. Recently, a novel mutation in *hVAPB* (V234I) has been identified but its pathogenic potential has not been assessed. We found that neuronal expression of the V234I mutant allele in *Drosophila* (*DVAP-V260I*) induces defects in synaptic structure and microtubule architecture that are opposite to those associated with *DVAP* mutants and transgenic expression of other ALS-linked alleles. Expression of *DVAP-V260I* also induces aggregate formation, reduced viability, wing postural defects, abnormal locomotion behavior, nuclear abnormalities, neurodegeneration and upregulation of the heat-shock-mediated stress response. Similar, albeit milder, phenotypes are associated with the overexpression of the wild-type protein. These data show that overexpressing the wild-type DVAP is sufficient to induce the disease and that *DVAP-V260I* is a pathogenic allele with increased wild-type activity. We propose that a combination of gain- and loss-of-function mechanisms is responsible for VAPB-induced ALS.

## Introduction

Amyotrophic Lateral Sclerosis (ALS) is a progressive neurodegenerative disorder characterized by the degeneration of motor neurons leading to muscle atrophy, spasticity and, eventually, death. ALS is traditionally classified into two categories: familial ALS (FALS) and sporadic ALS (SALS) (Pasinelli and Brown, 2006). Missense mutations (P56S and T46I) at the N-terminal region of the human VAMP-associated protein B gene (hVAPB) have been identified in both patients with FALS and SALS (Nishimura et al., 2004; Funke et al., 2010; Millecamps et al., 2010; Landers et al., 2008; Chen et al., 2010). VAP proteins contain an N-terminal domain, which is highly homologous to the nematode major sperm protein (MSP), a central domain that forms a coiled-coil structure and a C-terminal transmembrane domain (Nishimura et al., 1999; Kaiser et al., 2005).

Members of the highly conserved VAP family have been involved in a number of seemingly unrelated functions including regulation of ER morphology, lipid transfer, vesicular trafficking and dendritic morphogenesis (Soussan et al., 1999; Skehel et al., 2000; Amarilio et al., 2005; Teuling et al., 2007; Lev et al., 2008; Peretti et al., 2008; Kuijpers et al., 2013a). *DVAP*, the *Drosophila* orthologue of *hVAPB*, controls synaptic structure, microtubule cytoskeleton architecture and composition of post-synaptic glutamate receptors (Pennetta et al., 2002; Chai et al., 2008). In *C. elegans* and *Drosophila*, the MSP domain is cleaved, secreted and acts as a ligand for Robo and Lar-like receptors to control muscle mitochondria morphology, localization and function (Tsuda et al., 2008; Han et al., 2012). Previous studies have implicated ALS mutant alleles in an abnormal unfolded protein response and in the disruption of the anterograde axonal transport of mitochondria (Chen et al., 2010; Kanekura et al., 2006; Langou et al., 2010; Suzuki et al., 2009; Gkogkas et al., 2008; Mórotz et al., 2012). Transgenic expression in *Drosophila* of either *DVAP-P58S* or *DVAP-T48I*, two ALS-linked alleles, mirrors major hallmarks of the human disease including neurodegeneration, aggregate formation, locomotion defects and chaperone upregulation (Chai et al., 2008; Chen et al., 2010). Several lines of evidence support the notion that P56S mutation is a loss-of-function mutation, possibly *via* a dominant negative effect. P56S mutant protein forms aggregates in which the wild-type protein is recruited (Teuling et al., 2007). This together with the analysis of mutant phenotypes associated with transgenic expression of the P56S protein in several disease models, confirms that P56S is a loss-of-function mutation (Teuling et al., 2007; Chen et al., 2010; Chai et al., 2008; Ratnaparkhi et al., 2008; Tsuda et al., 2008; Suzuki et al., 2009; Forrest et al., 2013; Kuijpers et al., 2013a). Furthermore, a reduction in VAP protein levels has been reported in sporadic ALS patients, SOD1 (Superoxide dismutase 1) mutant mice as well as in induced pluripotent stem cells derived from ALS patients (Anagnostou et al., 2010; Teuling et al., 2007; Mitne-Neto et al., 2011). Recently, a novel mutation in the gene *hVAPB* was identified in one ALS patient who has also a pathogenic repeat expansion in *C9ORF72* (chromosome 9 open reading frame 72), another ALS causative gene. The mutation within the hVAPB gene replaces the Valine at the position 234 of the highly conserved transmembrane domain with an Isoleucine (V234I in humans and V260I in *Drosophila*) (van Blitterswijk et al., 2012). Moreover, no information is available about the dominant or recessive inheritance of the mutation in humans and theoretical predictions based on bioinformatics approaches give contradictory results as to whether V234I mutation has a damaging effect on the function of the protein (van Blitterswijk et al., 2012; Ingre et al., 2013). To directly assess the pathogenic effect of this mutation and to unveil potential new mechanisms of disease pathogenesis, we carried out a series of functional studies in a well-established *Drosophila* model of ALS.

Here we report that transgenic expression of *DVAP-V260I* recapitulates major hallmarks of the human disease including aggregate formation, reduced viability, neuromuscular defects, abnormal locomotion behavior, neurodegeneration and upregulation of the heat-shock-mediated stress response. Moreover, we show that nuclear abnormalities represent a novel aspect of ALS pathogenesis as expression of *DVAP-V260I* either in neurons or muscles induces disruption in nuclear architecture, position and shape. Surprisingly, we found that transgenic expression of *DVAP-V260I* at the *Drosophila* larval neuromuscular junction (NMJ) induces an increase in the number of synaptic boutons and a decrease in their size. This phenotype is highly reminiscent of the phenotype associated with the neuronal overexpression of DVAP-WT protein and opposite to that of DVAP loss-of-function mutations and transgenic expression of the *DVAP-P58S* allele in neurons (Pennetta et al., 2002; Forrest et al., 2013). In addition, overexpression of DVAP-WT protein either in neurons or muscles induces phenotypes similar, albeit milder, than those associated with V260I expression. Altogether these data lead to the fundamental conclusion that *DVAP-V260I* is a pathogenic allele with an increased wild-type activity and that a combination of loss- and gain-of-function mechanisms are responsible for VAP-induced ALS. In conclusion, on the basis of the data reported here and those on previously identified mutations, we propose that VAPB levels or/and activity must be tightly regulated to keep neurons and muscles healthy as slight disturbances in one direction or the other may induce cell dysfunction and death.

## Results

### Presynaptic expression of either DVAP-V260I or DVAP-WT transgenes leads to an overproduction of small synaptic boutons

The V234I mutation in the *hVAPB* gene recently identified in one ALS patient but not in a large number of healthy controls, is located within the conserved and functionally important transmembrane domain of VAP proteins (van Blitterswijk et al., 2012) (supplementary material Fig. S1). Conversely, the previously identified mutations, P56S and T46I, are localized in a highly conserved stretch of amino acids within the MSP domain (supplementary material Fig. S1C).

**Fig. 1.**
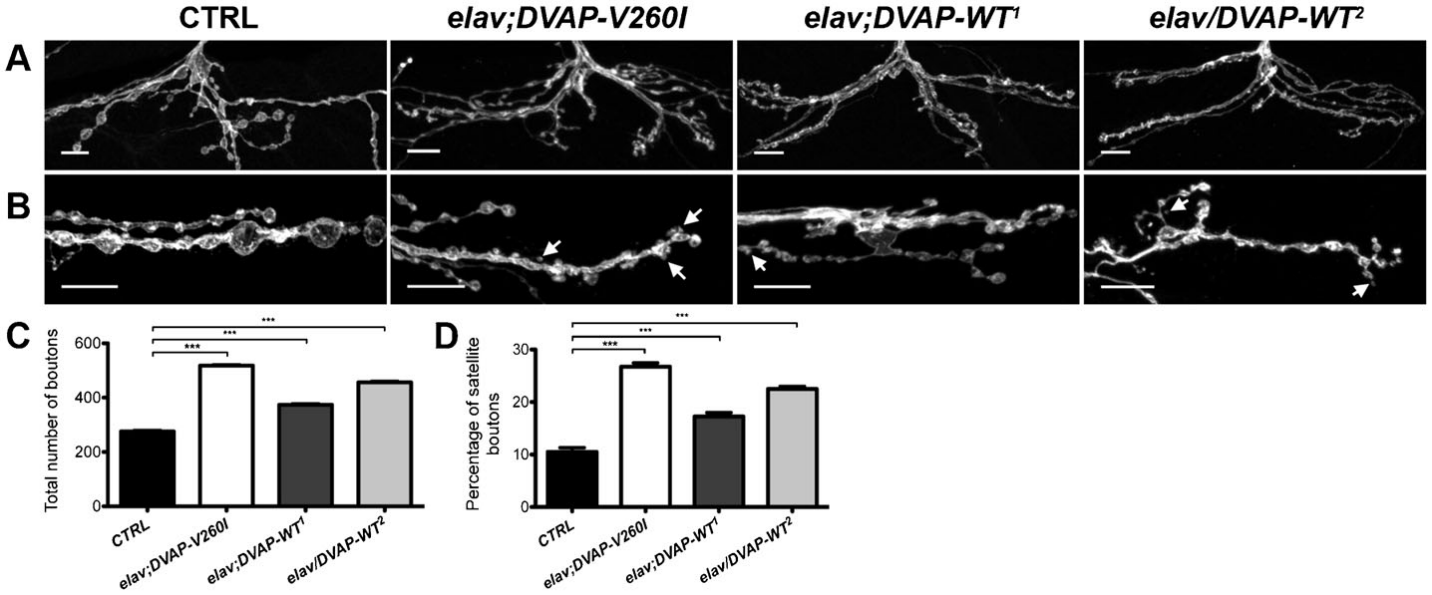
Synaptic boutons are smaller, more numerous and clustered at NMJs expressing either *DVAP-V260I* or *DVAP-WT* transgenes. (A) Representative images of muscle 12 NMJs from abdominal segment 3 labeled with an antibody against HRP. Scale bars: 10 µm. (B) Illustration of the synaptic overgrowth phenotype at HRP-stained NMJs of the indicated genotypes. In all panels arrows indicate satellite boutons. Scale bars: 10 µm. (C) Quantification of the total number of boutons at muscles 12 and 13 of abdominal segment 3 and (D) quantification of satellite boutons at muscles 6/7 of the abdominal segment 3 for each indicated genotype. *elav-Gal4/+* NMJs were used as controls. Asterisks denote statistical significance compared to controls (***P<0.001).

Presynaptic expression of the ALS-linked allele *DVAP-P58S* induces a NMJ phenotype characterized by a decrease in the number of boutons and an increase in their size (Chai et al., 2008; Ratnaparkhi et al., 2008). These data prompted us to examine the effect of DVAP-V260I expression on bouton formation and synaptic structure. We generated several transgenic lines expressing *DVAP-V260I* using the bipartite *UAS/GAL4* system and the panneural *elav-Gal4* as a driver (Lin and Goodman, 1994). To examine basic synaptic morphology, NMJs were stained with anti-HRP antibodies that label and allow for visualization of the entire presynaptic membrane. Changes in NMJ structure were assessed by counting the number of synaptic boutons at muscles 12 and 13 of the abdominal segment A3. This analysis revealed that transgenic expression of *DVAP-V260I* has clear consequences on the expansion of boutons as compared to controls. We were surprised to observe that, in this case, there is an increase in the total number of synaptic boutons when compared to controls (514±3 in *elav;DVAP-V260I* versus 272±2 in controls; P<0.001) (Fig. 1A,C). The size of boutons, however, is dramatically reduced.

We have previously shown that an increase in the number of boutons with a concomitant decrease in their size is associated with the presynaptic overexpression of DVAP-WT protein and that this phenotype is highly dependent on the dosage of the DVAP (Pennetta et al., 2002). To further confirm and extend this analysis, we generated a number of additional transgenic lines expressing different levels of the DVAP-WT protein and found that they all exhibited similar synaptic phenotypes. We selected a weak line (DVAP-WT^1^) in which the number of boutons on the muscles 12 and 13 of the segment A3 is 1.3-fold higher than in controls and a strong DVAP-WT^2^ line that exhibits a 1.7-fold increase in the number of synaptic boutons (Fig. 1A,C; P<0.001 in both cases).

We also noticed that in all transgenic lines the overall morphology of the synapse has changed. In control NMJs, synaptic boutons within a branch, resemble a string of beads with boutons connected to one another by a short neuritic process (Fig. 1B). In contrast, in larvae overexpressing either *DVAP-V260I* or *DVAP-WT* transgenes, many small boutons appear to bud off from a central bouton or from neuronal processes connecting two boutons (Fig. 1B, arrows). Clusters of smaller boutons surrounding central larger boutons are especially common in DVAP-V260I overexpressors (Fig. 1B,D). Boutons of similar size and morphology have been described in other overgrowth synaptic phenotypes and they have been called “satellite” boutons (Torroja et al., 1999; Franco et al., 2004; Kamimura et al., 2013). To correct for variations in total bouton number, we quantified the amount of “satellite” boutons for each genotype as a percentage of the mean total bouton number. We found that presynaptic expression of *DVAP-V260I* using the neuronal *elav-Gal4* driver, induces a 2.6-fold increase in the percentage of “satellite” boutons compared to controls (Fig. 1B,D; P<0.001). Neuronal expression of either *DVAP-WT^1^* or *DVAP-WT^2^* transgenes using the same Gal4 driver, leads to 1.7-fold and 2.2-fold increase in satellite bouton number, respectively (Fig. 1B,D; P<0.001 in both cases). In summary, expression of either *DVAP-WT* or *DVAP-V260I* transgenes induces a dramatic change on the overall morphology of the synapse due, not only, to an increase in the total number of boutons but also to an elevated number of “satellite” boutons.

It was surprising to find an overgrowth synaptic phenotype associated with *DVAP-V260I* as transgenic expression of the previously identified *DVAP-P58S* allele in neurons leads to an opposite phenotype characterized by a decrease in the number of boutons and an increase in their size (Pennetta et al., 2002; Chai et al., 2008). The *DVAP-P58S*-induced phenotype is similar to that associated with *DVAP* loss-of-function mutations (Pennetta et al., 2002) leading to the hypothesis that *DVAP-P58S* is a loss-of-function allele (Pennetta et al., 2002; Ratnaparkhi et al., 2008; Chai et al., 2008). Accordingly, we found that in DVAP-P58S transgenics the endogenous wild-type protein accumulates in the aggregates distributed along the nerves and is depleted from its normal synaptic localization (supplementary material Fig. S2E, arrow). In particular, quantification of DVAP-positive staining at NMJs of *DVAP-P58S* expressing neurons results in nearly 45% decrease in the DVAP immunoreactivity compared to controls (supplementary material Fig. S2A,E,F; P<0.001). We then assessed the distribution and the local expression levels of DVAP at NMJs of both the DVAP-V260I and the two DVAP-WT lines. In agreement with the observed synaptic phenotypes, we found that *elav-Gal4*-driven expression of every transgene results in a rather homogenous distribution of DVAP at the synapse and quantification of DVAP-positive immunoreactivity revealed that in every case the amount of total protein is higher than in controls (supplementary material Fig. S2). Specifically, the increase in DVAP signal is 2.2-fold higher than in controls for the DVAP-WT^2^ line while it is 1.7-fold and 1.8-fold over the endogenous levels for DVAP-V260I and DVAP-WT^1^, respectively (supplementary material Fig. S2, P<0.001 in all cases). It is worth to note that although DVAP-WT^1^ and DVAP-V260I lines express comparable amounts of their respective transgenes, expression of the DVAP-WT^1^ transgene leads to synaptic phenotypes and indeed to a number of other phenotypes (see below) that are milder than those associated with the transgenic expression of the mutant allele (compare supplementary material Fig. S2 and Fig. 1). Furthermore, phenotypes induced by the expression of *DVAP-V260I* in eight tested lines are consistently more severe than those observed in a comparable number of lines expressing the *DVAP-WT* transgene (data not shown). Taken together, these results indicate that an increase in the levels of DVAP wild-type protein is sufficient to induce an overgrowth synaptic phenotype and that the *DVAP-V260I* mutant allele acts as a hypermorphic allele as it has an increased ability to promote bouton formation at the NMJ.

### Presynaptic transgenic expression of *DVAP-WT* and *DVAP-V260I* leads to bouton expansion by affecting microtubule cytoskeleton architecture

Formation of synaptic boutons at axon terminals is achieved via microtubules invading and promoting bouton budding from the plasma membrane (Roos et al., 2000). Within the *Drosophila* NMJ, excess of microtubule loop formation is associated with overgrowth phenotypes, particularly those with an increased number of “satellite” boutons (Franco et al., 2004; Kamimura et al., 2013). Thus, we asked whether the increase in bouton number in NMJs expressing either *DVAP-WT* or *DVAP-V260I* transgenes is related to alterations in microtubule cytoskeleton architecture. To address this question, we carried out immunostainings against the *Drosophila* MAP1B homologue Futsch, a marker of neuronal microtubule structures (Hummel et al., 2000; Roos et al., 2000).

**Fig. 2.**
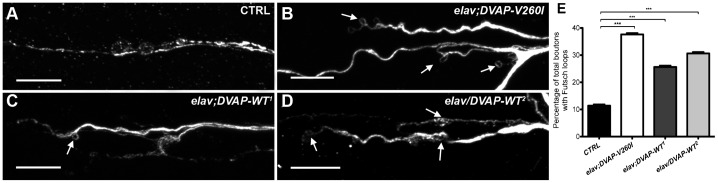
Expression of either *DVAP-V260I* or *DVAP-WT* transgenes in neurons affects synaptic microtubule cytoskeleton. (A) Representative images of branches of NMJs of third instar *elav-Gal4/+* control larvae, (B) *elav;DVAP-V260I*, (C) *elav;DVAP-WT^1^* and (D) *elav/DVAP-WT^2^* larval NMJs labeled with antibodies against Futsch to show microtubule loops. Arrows in every panel indicate examples of Futsch loops. Scale bars: 10 µm. (E) Quantitative assessment of Futsch-positive loops at A2 and A3 muscle 4 NMJs for each indicated genotype. The highest increase in the percentage of boutons exhibiting looped Futsch staining (relative to the total number of boutons for each NMJ) was observed when DVAP-V260I was expressed presynaptically. Asterisks denote statistical significance compared to controls (***P<0.001).

*DVAP-WT^1^*, *DVAP-WT^2^* and *DVAP-V260I* transgenes were each expressed in neurons using the *elav-Gal4* driver and the number of Futsch-positive microtubule loops was quantified for every genotype. Presynaptic overexpression of *DVAP-V260I* shows a drastic increase in the number of Futsch-positive loops as compared to controls (38±0.4% versus 11±0.4% in controls, P<0.001) (Fig. 2A,B,E). A significant increase in the proportion of boutons containing a microtubule loop-like structure was also observed following the presynaptic expression of *DVAP-WT* transgenes (26±0.5% for DVAP-WT^1^ and 31±0.5% for DVAP-WT^2^). However, in both cases, the change is less than that associated with *DVAP-V260I* overexpressing mutants (Fig. 2C,D,E). Of note, an increase in number of boutons with more disorganized microtubule structure represented by splayed/punctuate Futsch-positive immunostaining was reported in larvae expressing *DVAP-P58S* and in *DVAP* loss-of-function mutants (Pennetta et al., 2002; Forrest et al., 2013). As expected, these NMJs exhibit an opposite synaptic phenotype characterized by a decrease in number of boutons and an increase in their size (Pennetta et al., 2002; Forrest et al., 2013). Thus, expression of either *DVAP-V260I* or *DVAP-WT* transgenes in neurons, affects the organization of microtubule architecture, albeit at a different degree, with consequences on synaptic bouton formation and division.

### Targeted expression of either *DVAP-V260I* or *DVAP-WT* transgenes induces aggregate formation and nuclear abnormalities

DVAP is a ubiquitous protein and pathological alterations in muscles during ALS have been considered to result from toxicity of the mutant protein in motor neurons rather than a direct effect of the pathogenic protein on striated muscles. In order to explore whether the muscle is a direct target of DVAP-V260I and DVAP-WT toxicity, the expression of either the wild-type or mutant allele was selectively targeted to striated muscles, by using the muscle-specific driver *BG57-Gal4* (Budnik et al., 1996).

**Fig. 3.**
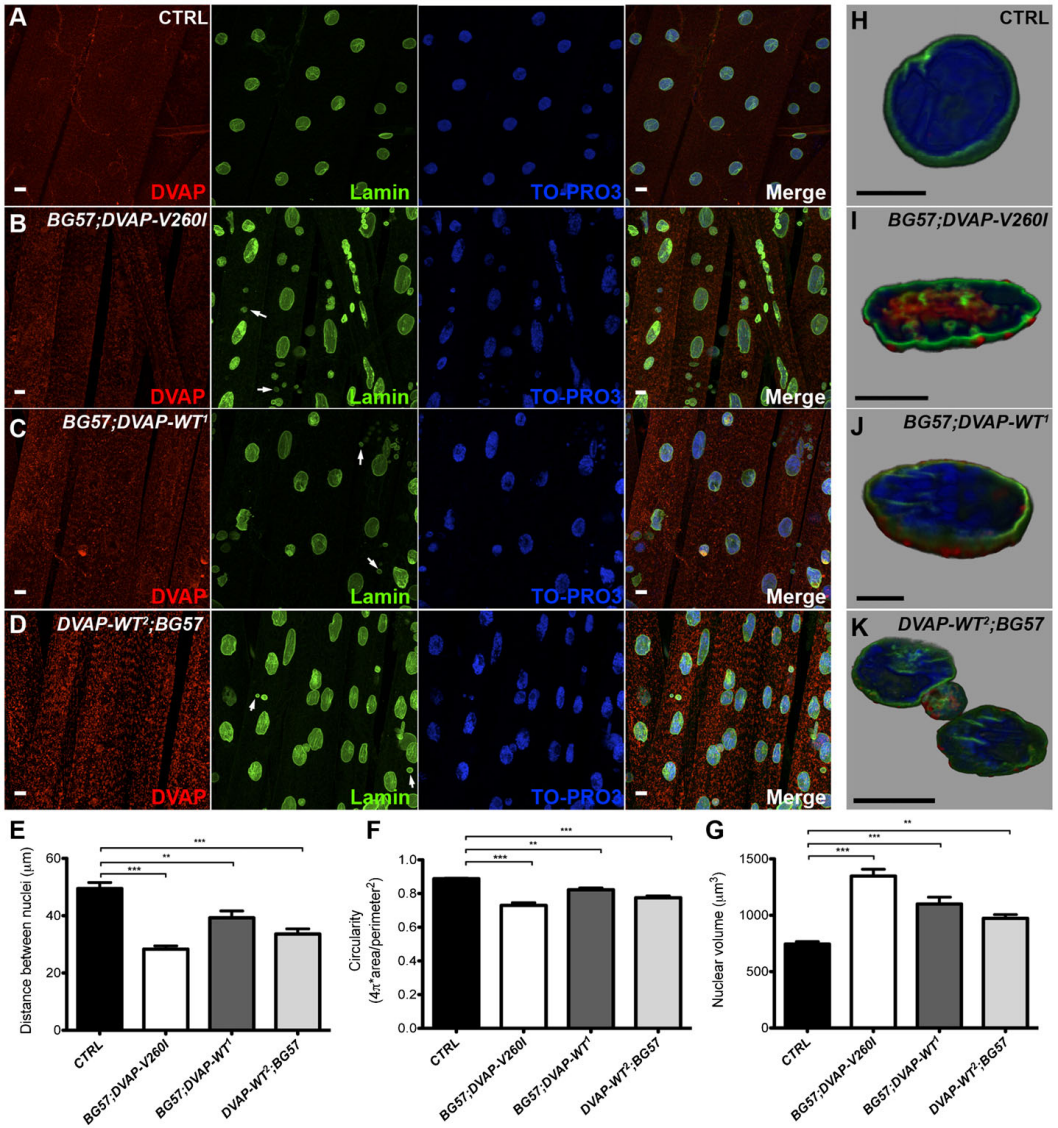
Postsynaptic expression of either *DVAP-V260I* or *DVAP-WT* transgenes results in aggregate formation and changes in nuclear shape, size and positioning. (A) Third instar larval NMJs of *BG57-Gal4/+* control, (B) *BG57;DVAP-V260I*, (C) *BG57;DVAP-WT^1^* and (D) *DVAP-WT^2^;BG57* NMJs expressing the indicated transgene in muscles were labeled with antibodies specific for DVAP and lamin. Nuclei were visualized with the nuclear specific marker TO-PRO3. (E) Quantification of the distance, (F) circularity and (G) volume of nuclei in randomly selected muscles for each indicated genotype. Sectioned volume renderings of representative triple-labelled nuclei of *BG57-Gal4/+* controls (H); (I) *BG57;DVAP-V260I*; (J) *BG57;DVAP-WT^1^* and *DVAP-WT^2^;BG57* (K). Scale bars: 10 µm. Asterisks denote statistical significance. ***P<0.001, **P<0.01.

Abnormal aggregates containing disease proteins are a common hallmark of several neurodegenerative disorders. We therefore stained dissected NMJs with DVAP antibodies to assess the localization of DVAP proteins in controls and in muscles expressing either the *DVAP-WT* or the *DVAP-V260I* transgenes. We found that while in controls DVAP is expressed throughout the muscle, in DVAP-V260I muscles numerous and punctuate, DVAP-immunopositive inclusions are present (Fig. 3A,B, Fig. 5A,B, staining in red). Interestingly, similar inclusions are also found in both the weak (DVAP-WT^1^) and strong (DVAP-WT^2^) transgenic lines overexpressing the DVAP-WT protein (Fig. 3C,D, Fig. 5C,D, staining in red). These data indicate that overexpression of the DVAP-WT protein is sufficient to induce the formation of aggregates and that *DVAP-V260I* is a pathogenic allele.

Nuclear defects have been correlated with ageing as well as with a number of pathological manifestations in humans including Parkinson's disease (Worman et al., 2010; Liu et al., 2012). To determine whether nuclear architecture or/and positioning are affected in our ALS fly model, we visualized nuclei in muscles overexpressing either *DVAP-V260I* or *DVAP-WT* transgenes using a nuclear marker and an anti-lamin antibody. We found that in controls, nuclei are evenly spaced along the muscle fibre while in muscles expressing either the *DVAP-V260I* or the *DVAP-WT* transgenes, nuclei are closely associated and exhibit a tendency to form clusters (Fig. 3). To better evaluate the relative position of nuclei within a muscle, a “nearest-neighbor” analysis was conducted for each genotype (see Materials and Methods for details). We found that, compared to controls, the average shortest distance between nuclei is severely reduced in muscles overexpressing the *DVAP-V260I* transgene (P<0.001) while the overexpression of the *DVAP-WT* transgenes has a significant but milder effect even in the case of the strongest DVAP-WT^2^ line (Fig. 3E; P<0.01 for DVAP-WT^1^ and P<0.001 for DVAP-WT^2^).

We also noticed a drastic deterioration in nuclear architecture. In particular, overexpression of either *DVAP-WT* or *DVAP-V260I* transgenes results in deformed nuclei with an elongated structure. To quantify this phenotype, we measured the width to length ratio of nuclei within muscles per each genotype. As the nuclei in control muscles have a distinct round shape, the width to length ratio is close to 1. Any deviation from this value indicates a loss of circularity and therefore a change in shape. We found that nuclei within muscles overexpressing the *DVAP-V260I* transgene are less round compared to nuclei in control muscles (Fig. 3A,B,F; P<0.001) and a similar, albeit milder effect, was observed in muscles overexpressing the *DVAP-WT* transgenes (Fig. 3A,C,D,F; P<0.01 for DVAP-WT^1^ and P<0.001 for DVAP-WT^2^). We also found that nuclei in muscles expressing the *DVAP-V260I* transgene display a marked enlarged nuclear volume compared to controls (Fig. 3G; P<0.001) as do the nuclei of muscles expressing the *DVAP-WT* transgenes, although to a lesser extent (Fig. 3G; P<0.001 for the DVAP-WT^1^ and P<0.01 for DVAP-WT^2^). Intriguingly, a fraction of DVAP protein that is predominantly a cytoplasmic protein was found to localize to the nucleus in *DVAP-V260I* overexpressing muscles while in both DVAP-WT lines this aberrant DVAP localization is nearly absent (Fig. 3H–K). A nuclear localization for DVAP has never been described before but previous studies have shown that translocation from the cytoplasm to the nucleus of α-synuclein associates with increased toxicity and neurodegeneration in Parkinson's disease (Kontopoulos et al., 2006). Moreover, an increased number of small and condensed pyknotic nuclei was predominantly observed in muscles overexpressing the *DVAP-V260I* transgene while such nuclei are significantly less in the weak DVAP-WT^1^ line (Fig. 3, white arrows).

**Fig. 4.**
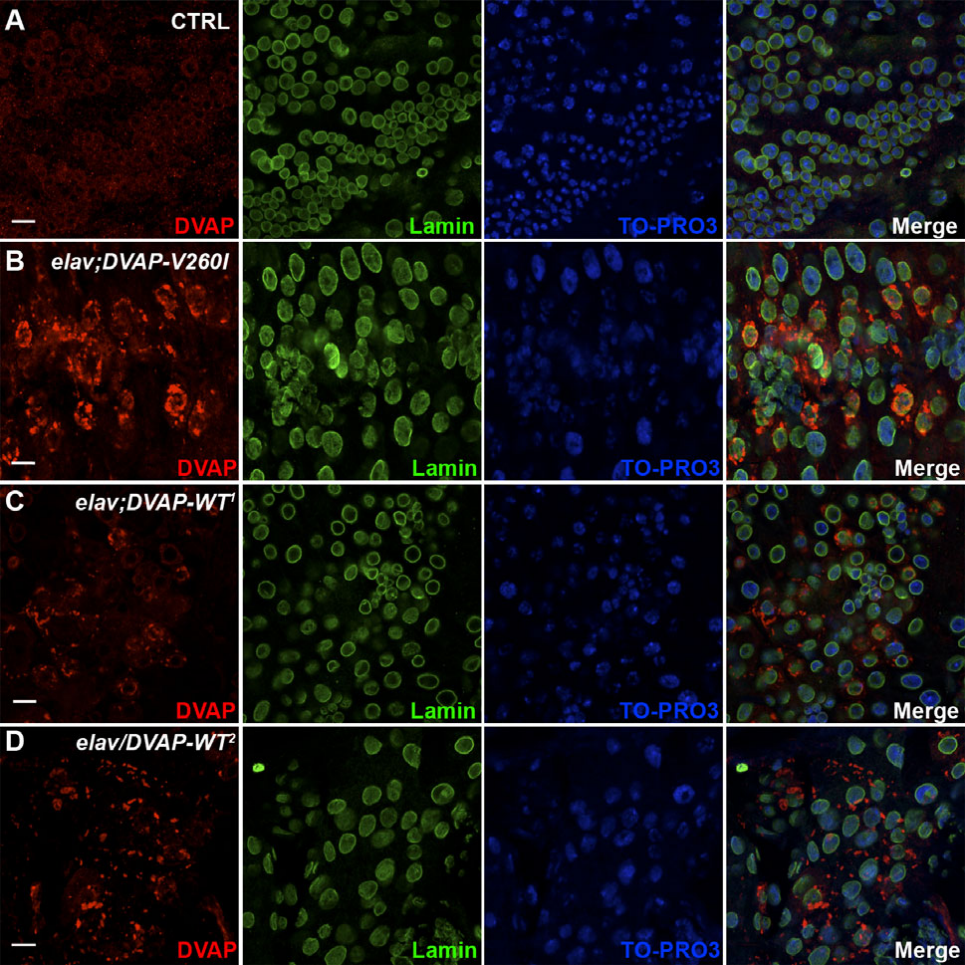
Expression of *DVAP-V260I* and *DVAP-WT* transgenes in neurons leads to aggregate accumulation and disruption of nuclear architecture. (A) Brains of *elav-Gal4/+* control larvae and (B) *elav;DVAP-V260I*, (C) *elav;DVAP-WT^1^*, (D) *elav/DVAP-WT^2^* larval brains expressing their respective transgene in neurons were immunostained with antibodies specific for DVAP and Lamin. Nuclei were labeled with the nuclear marker TO-PRO3. Scale bars: 10 µm.

Finally, we noticed that larger and malformed nuclei are also observed in neurons of larval brains expressing either the *DVAP-WT* or *DVAP-V260I* transgenes and again the phenotype appears to be more severe when the mutant transgene is expressed (Fig. 4). In addition, expression of *DVAP-V260I* in neurons also induces accumulation of prominent inclusions that appear to be very large and intensely immunoreactive to DVAP antibodies (Fig. 4A,B). In contrast, in neurons of the weakest DVAP line (DVAP-WT^1^), small and isolated foci accumulating DVAP were observed. Although not directly quantified, the number of these foci appear to be increased in neurons of the strongest *DVAP-WT^2^* line (Fig. 4C,D). Interestingly, no small pyknotic nuclei were observed in these neurons that present an aberrant nuclear architecture.

Taken together, these data demonstrate that transgenic expression of either *DVAP-V260I* or *DVAP-WT* in neurons as well as in muscles elicits the formation of aggregates and a severe disruption in the architecture, size and positioning of nuclei.

### Induction of the heat-shock response in DVAP-WT and DVAP-V260I overexpressors

**Fig. 5.**
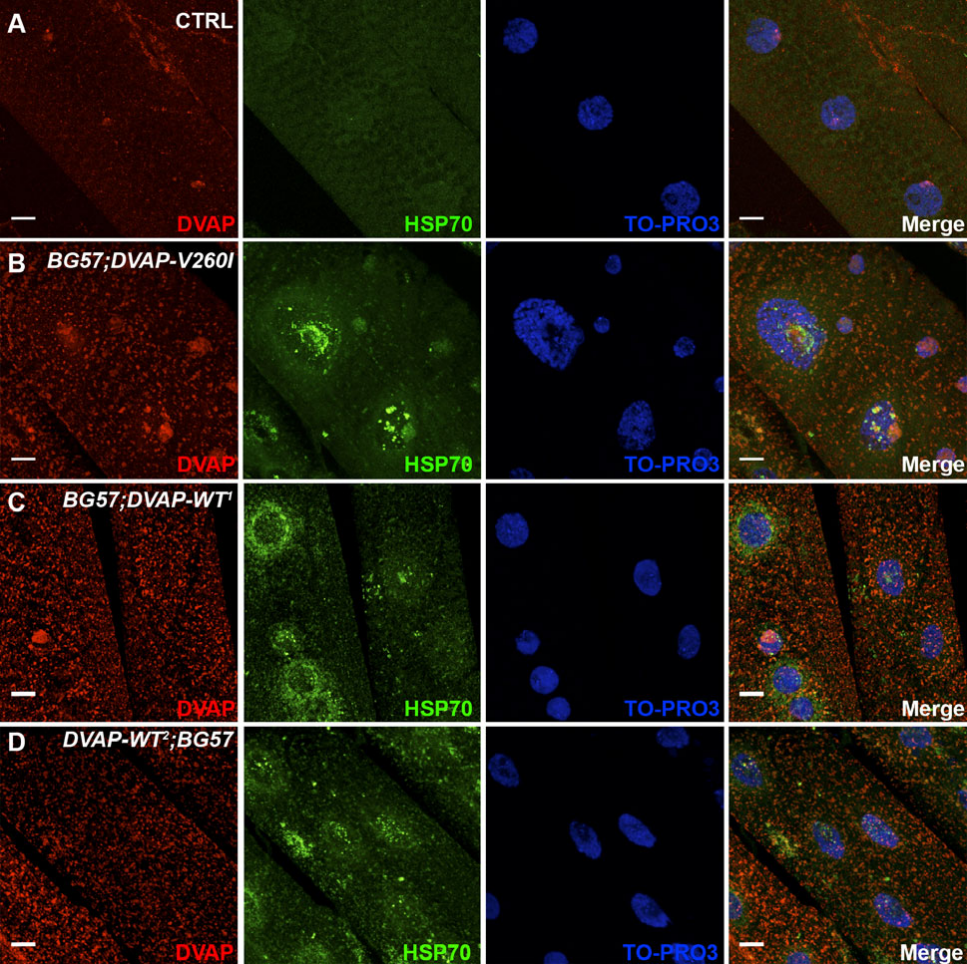
Upregulation and subcellular relocalization of Hsp70 in striated muscles overexpressing either *DVAP-V260I* or *DVAP-WT* constructs. (A) NMJs of *BG57-Gal4/+* control larvae and (B) *BG57;DVAP-V260I*, (C) *BG57;DVAP-WT^1^* and (D) *DVAP-WT^2^;BG57* larvae expressing their respective transgene were immunolabeled with antibodies specific for DVAP and Hsp70 while nuclei were visualized with the TO-PRO3 nuclear marker. Scale bars: 10 µm. See text for comments.

In many neurodegenerative diseases including ALS, aggregates are thought to be formed from the accumulation of misfolded proteins that trigger the upregulation of chaperone proteins (Vabulas et al., 2010). We assessed whether this was also the case for larvae expressing either *DVAP-V260I* or *DVAP-WT* transgenes in their muscles. In controls, Hsp70 is barely detectable and results in faint and diffuse staining throughout the cytoplasm (Fig. 5A). In transgenic animals expressing the *DVAP-V260I* construct specifically in the striated muscles, there is an increase in the expression levels of Hsp70 that appears to concentrate in puncta mainly localized inside the nucleus (Fig. 5B). In transgenic muscles expressing the *DVAP-WT* transgenes, an increase in the accumulation of puncta immunoreactive to the Hsp70 was observed. These puncta are partially localized into the nucleus in muscles expressing the strongest wild-type transgene *DVAP-WT^2^* (Fig. 5D). Conversely, in muscles expressing the weak DVAP-WT^1^ transgene, puncta are still present in the cytoplasm, although the majority of them preferentially accumulate in the perinuclear region (Fig. 5C). The phenomenon of the Hsp70 relocating from the cytoplasm to the nucleus during a stress response is poorly understood; however, it is clear that this relocation is directly linked and is part of the cellular response to stress (Vabulas et al., 2010). Taken together, these data indicate that in DVAP-V260I as well as in DVAP-WT overexpressors, a stress response mediated by heat-shock proteins has been induced and that the intensity of this response directly correlates with the toxicity of the overexpressed protein.

### Expression of DVAP-V260I and DVAP-WT in adult flies induces reduced viability, compromised locomotion behavior and neuromuscular defects

**Fig. 6.**
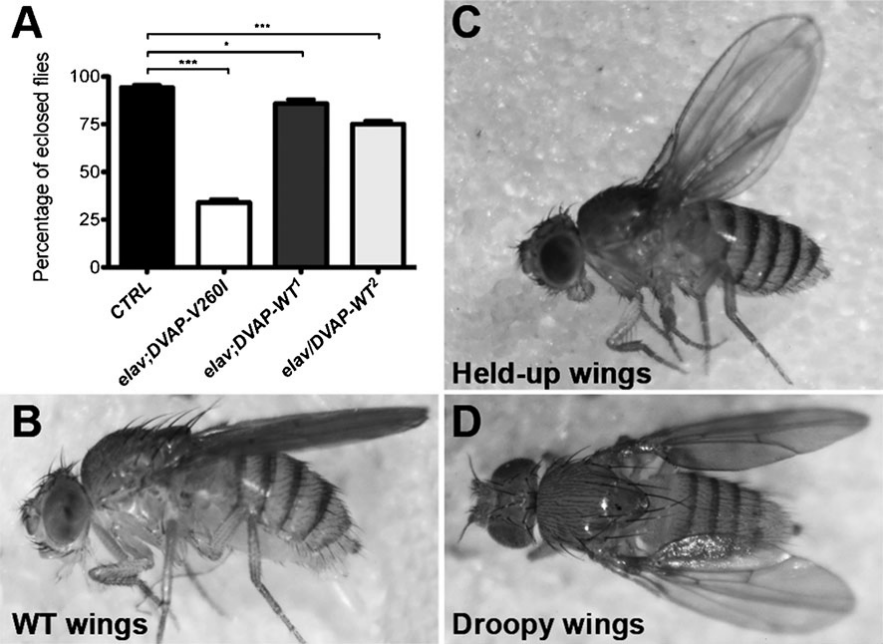
*DVAP-V260I* and *DVAP-WT* overexpressors display reduced viability and wing postural defects. (A) Eclosion rate of flies of the designated genotype. (B) *elav-Gal4/+* control flies with a dorsal wing posture. (C) Held-up wings and (D) droopy wing flies. See text for comments. Asterisks denote statistical significance. ***P<0.001, *P<0.05.

We showed that expression of *DVAP-V260I* and *DVAP-WT* transgenes in larval neurons and muscles causes a number of phenotypes closely mirroring the human pathology. We then sought to test whether the same transgenes have any effect on the adult neuromuscular junctions. We first expressed the *DVAP-V260I* or the *DVAP-WT* transgenes in the adult nervous system by using the panneural driver *elav-Gal4*. Flies expressing either the *DVAP-V260I* transgene or any of the two wild-type transgenic construct, are able to pupate and adults eclose from pupae. However, the rate of eclosion is different depending on the overexpressed transgene (Fig. 6A). Flies expressing the DVAP-WT^1^ transgene have a small but statistically significant decrease in the rate of eclosion (Fig. 6A; P<0.05) while for flies expressing the strongest wild-type allele, DVAP-WT^2^, the rate of eclosion is 75% (Fig. 6A; P<0.001). A strong reduction in the number of flies eclosed as viable adults is observed when the expression of the *DVAP-V260I* was targeted in the adult neurons. In this case only 35% of pupae eclose to give an adult fly (Fig. 6A). However, all flies, irrespective of their genotypes, display distinctive postural and locomotion defects as viable adults. While in controls, wings run dorsal and parallel to the body, surviving mutants exhibit different wing posture phenotypes including droopy and held-up wings (Fig. 6B–D). Additionally, surviving adult flies are severely uncoordinated and within a few days following eclosion, get stuck to the food and die. When the expression of either *DVAP-WT* or *DVAP-V260I* constructs is targeted to all muscles by using the *MHC-Gal4* driver, the progeny exhibits the same wing posture phenotypes as for the neuronal expression of the same transgenes but their eclosion ratio is similar for every transgene and closer to that of controls (supplementary material Fig. S3). Similar adult phenotypes have been described in a number of fly models of human neurodegenerative diseases including Fragile X syndrome and Parkinson's disease and have been attributed to neuromuscular dysfunction (Zhang et al., 2001; Clark et al., 2006).

### Targeted expression of either *DVAP-WT* or *DVAP-V260I* transgenes in the adult *Drosophila* eye causes neuronal degeneration

**Fig. 7.**
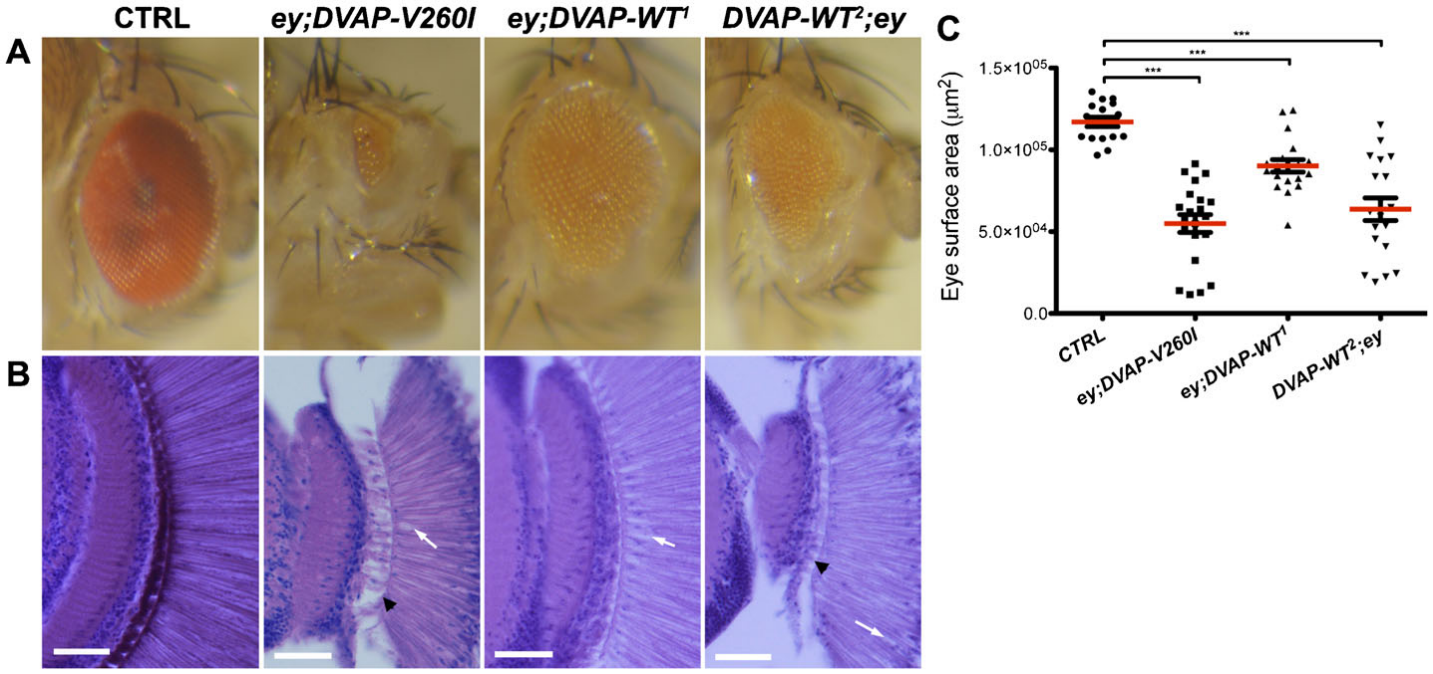
Expression of either *DVAP-V260I* or *DVAP-WT* transgenes in adult *Drosophila* eyes induce neurodegeneration. (A) Stereomicroscope images of *ey-Gal4/+* control eyes and eyes of the indicated genotypes. (B) Frontal sections of control and transgenic eyes of the indicated genotypes stained with H&E. White arrows point to vacuoles in photoreceptors while the black arrowheads indicate areas of extensive tissue degeneration. (C) Quantification of the eye surface area of every genotype. Scale bars: 50 µm. Asterisks denote statistical significance (***P<0.001).

Neuronal degeneration in *Drosophila* models of human neurodegenerative disorders including ALS is usually assessed in the adult fly eye. To determine whether overexpression of DVAP-WT protein and transgenic expression of DVAP-V260I causes neurodegeneration, we used the *ey-Gal4* driver to specifically target the expression of these transgenes in the adult fly eye (Halder et al., 1998; Hauck et al., 1999). Adult fly eyes expressing either *DVAP-WT* or *DVAP-V260I* transgenes display a range of structural abnormalities. As compared with controls, eyes expressing either the *DVAP-WT* or *DVAP-V260I* transgenes are reduced in size and show a marked roughness over the entire surface (Fig. 7A). A striking aspect of the eye phenotype induced by the overexpression of either DVAP-WT or the DVAP-V260I transgenes, is the high degree of heterogeneity in the severity of the phenotype within the same line and even within the same fly as, in many cases, one of the eyes exhibits a more extensive degeneration than the eye on the opposite side. We therefore quantified the phenotype in both control and overexpressor lines by measuring the eye size in a comparable number of flies for every genotype. Overall, the eye size of DVAP-V260I transgenic flies is less than 50% of the control size while the measured reduction in eye size for DVAP-WT^1^ and DVAP-WT^2^ was 30% and 20%, respectively (Fig. 7C; P<0.001 in all cases).

Histopathological analysis of paraffin embedded sections of transgenic eyes expressing either *DVAP-WT* or *DVAP-V260I* confirmed the degeneration of cells beneath the external surface of the eye (Fig. 7B). In particular, photoreceptor morphology is disrupted and several vacuoles are visible. Vacuolization has been observed in a number of fly models of neurodegenerative diseases and the appearance of vacuoles has been correlated to neurodegeneration (Chan and Bonini, 2000). Of note, the strongest phenotype for both the reduction in the eye size and the degeneration of the internal eye is that associated with the expression of the *DVAP-V260I* transgene (Fig. 7). The internal tissues of transgenic eyes expressing the DVAP-WT protein are disrupted in a similar way, albeit to a lesser extent, especially when the weakest line DVAP-WT^1^ is considered (Fig. 7B). In conclusion, these data indicate that increasing the level of expression of the DVAP-WT protein is sufficient to trigger neurodegeneration, although transgenic expression of the mutant *DVAP-V260I* allele appears to be more efficient in inducing neuronal cell death.

## Discussion

Recently, a novel mutation in the gene *hVAPB* has been identified in one ALS patient who is also known to have a pathogenic repeat expansion in the *C9ORF72* gene. The missense mutation replaces the Valine with an Isoleucine at codon 234, a highly conserved residue within the C-terminal transmembrane domain of VAP proteins (van Blitterswijk et al., 2012). In *Drosophila*, the transmembrane domain of DVAP binds the phosphoinositide phosphatase Sac1 to control phosphoinositide levels whose upregulation is responsible for neurodegeneration and a number of synaptic phenotypes including altered synaptic morphology and mislocalization of several post-synaptic markers (Forrest et al., 2013). Moreover, in mammals the transmembrane-mediated interaction of VAPB with the ER–Golgi recycling protein YIF1A controls dendritic remodeling in the central nervous system (Kuijpers et al., 2013a). It is therefore plausible that mutations targeting this important functional domain may have a pathogenic effect.

Here we report that transgenic expression of *DVAP-V260I* in *Drosophila*, induces mutant phenotypes that closely mirror major hallmarks of ALS such as structural remodeling at NMJs, formation of aggregates, abnormal locomotion behavior, reduced viability, neuromuscular defects and upregulation of the heat-shock-mediated stress response. In addition, muscles and neurons expressing the *DVAP-V260I* transgene exhibit striking abnormalities in nuclear positioning, shape and size. Interestingly, nuclear abnormalities have never been linked to ALS pathogenesis.

Previously identified disease mutations in *hVAPB* are loss-of-function mutations, possibly by a dominant negative mechanism (Ratnaparkhi et al., 2008; Forrest et al., 2013; Kuijpers et al., 2013a). We were therefore surprised to find that transgenic expression of *DVAP-V260I* construct consistently produces similar but more severe phenotypes than those associated with the overexpression of the wild-type protein. Collectively, our results show that a gain-of-function mechanism is responsible for VAPB-induced ALS and that the V234I is a pathogenic allele with an increased wild-type activity. In conclusion, we propose that both gain- and loss-of-function effects play an important role in driving the damage, which ultimately leads to cellular downfall and clinical expression of the disease.

### Nuclear abnormalities in ALS pathogenesis

Mispositioning of nuclei has been associated with decreased cell survival in vertebrate central nervous system (Tsujikawa et al., 2007). In striated muscles, myonuclei appear as rounded structures evenly spaced along muscle fibers separated from the bulk of the cytoplasm, which contains densely arranged myofibrils. The mechanisms establishing and maintaining this highly ordered distribution of nuclei and its relevance to muscle function is not known but several human myopathies are caused by mutations in genes such as Nesprins that control the correct position and distribution of nuclei in muscles (Puckelwartz et al., 2010). Furthermore, Nesprin-1 knockout mice exhibit 50% lethality and those surviving display progressive muscle wasting and abnormal gait (Puckelwartz et al., 2009). Nuclear architecture defects have been shown to correlate with aging as well as with a number of human diseases, including Parkinson's disease (Liu et al., 2012).

To study the role that nuclear position and structure might have in ALS, we targeted the expression of *DVAP-WT* and *DVAP-V260I* in neurons and muscles. Our morphometric analysis revealed that overexpression of the *DVAP-V260I* results in deformed nuclei with an elongated structure and a marked enlarged nuclear volume. We also found that in *DVAP-V260I* transgenic muscles the average distance between nuclei is remarkably reduced and sometimes, nuclei are found to aggregate into clusters. A similar deterioration in nuclear architecture and positioning was observed in muscles overexpressing *DVAP-WT* transgenes but, overall, the defects are milder than those associated with the expression of *DVAP-V260I* even when the strongest *DVAP-WT^2^* transgene is expressed. In summary, nuclear abnormalities are more prominent in transgenic conditions associated with a more aggressive disease phenotype.

Finally, small and condensed pyknotic nuclei were observed in muscles expressing *DVAP-V260I* and to a lesser extent in those overexpressing the *DVAP-WT* transgene. Although the presence of pyknotic nuclei is a hallmark of apoptosis, we do not believe that the observed nuclear abnormalities are a mere consequence of apoptotic cell death. No condensed, pyknotic nuclei are present in neurons expressing any *DVAP-WT* transgenes or DVAP-V260I protein at the time when nuclear abnormalities are already visible. These results suggest that defects in nuclear architecture and distribution may precede the appearance of apoptotic hallmarks. Indeed recent studies suggest that alterations in nuclear structure and positioning represent an irreversible trigger of apoptosis rather than a consequence of this process (Liu et al., 2012).

Taken together, these studies identify the nucleus as a novel cellular organelle involved in ALS pathogenesis and open new avenues for the potential development of therapies targeting this fundamental cellular structure.

### Expression of either *DVAP-V260I* or *DVAP-WT* transgenes recapitulates major ALS-related hallmarks

The data discussed in the previous paragraph suggest that our DVAP-V260I fly model may function as a platform for the discovery and study of novel and, at the moment, elusive aspects of ALS pathogenesis. Our data also suggest that this model recapitulates many of the phenotypes known to be associated with ALS and indeed with a number of other neurodegenerative diseases.

In *Drosophila*, DVAP interacts with the adhesion molecule Dscam to affect synaptic connectivity and its homologue in mammals, VAPB, is required for intracellular membrane trafficking and normal dendritic growth (Yang et al., 2012; Kuijpers et al., 2013a). In addition, loss-of-function mutations in the SMN (Survival motor neuron) gene, the causative gene of the motor neurone disease spinal muscular atrophy (SMA), induces defects in axons and synaptic structure (Torres-Benito et al., 2012). Similar results have been reported for the ALS-linked gene VCP (Valosin Containing Protein) as mutations in this gene lead to impaired synaptogenesis and spinogenesis (Wang et al., 2011). To study the effect of *DVAP-WT* and mutant *DVAP-V260I* transgenes on synaptic remodeling and structure, we focused on the *Drosophila* larval NMJ. Morphological analysis shows that at NMJs overexpressing either *DVAP-WT* or *DVAP-V260I* transgenes, boutons are smaller, more numerous and tend to form dense clusters especially at the ends of terminal branches. Overgrowth synaptic phenotypes have been associated with alterations in microtubule networks and specifically with an increase in the percentage of boutons containing microtubule loop-like structures (Torroja et al., 1999). Indeed we found that at NMJs expressing DVAP-V260I there is a specific increase in this category of boutons. Similar, but less severe, phenotypes are associated with the transgenic expression of the DVAP-WT protein. In contrast, expression of the ALS-linked allele *DVAP-P58S* in neurons and loss-of-function mutations in DVAP induce a reduction in number of boutons and an increase in their size. A shift in microtubule structures towards less organized morphologies, which is often associated with undergrowth synaptic phenotypes, was also observed in this transgenic condition (Pennetta et al., 2002). In agreement with these data, we show that there is a local increase in DVAP levels at DVAP-V260I expressing NMJs and a decrease in synapses expressing DVAP-P58S. In summary, we propose that disruption of synaptic remodeling and structure may represent a fundamental and common aspect of disease pathogenesis in ALS and that the *DVAP-V260I* allele, in contrast to the *DVAP-P58S* allele, exhibits an increased wild-type activity.

A common feature of neurodegenerative diseases is the presence of misfolded protein aggregates in affected regions of the nervous system. Many neurodegenerative proteins behave like prion proteins (Polymenidou and Cleveland, 2012). *In vivo* and *in vitro* studies have shown that these proteins exhibit a high tendency to self-aggregate because of their intrinsic ability to undergo a series of kinetically unfavorable conformational changes to produce misfolded proteins. Increasing the concentration of the native protein favors the formation of aggregates and disease-linked mutations often appear to confer to the protein an increased ability to generate inclusions (Eisele, 2013). Further studies are necessary to assess whether DVAP is a prion-like protein. However, as expected for a prion-like protein, DVAP-WT overexpression is sufficient to induce the formation of aggregates in a dosage-dependent manner and transgenic expression of the mutant DVAP-V260I protein effectively leads to the accumulation of large protein inclusions even at relatively low doses.

In our *Drosophila* model, muscles expressing the *DVAP-V260I* transgene, which associates with the most aggressive disease phenotype, exhibit a relocalization of DVAP protein into the nucleus. DVAP remains predominantly localized to the cytoplasm in controls and in all the other transgenic conditions exhibiting a milder disease phenotype. The mechanism by which DVAP enters the nucleus is unclear; however, this relocalization closely resembles the relocalization from the cytoplasm to the nucleus reported for α-synuclein, a protein involved in Parkinson's disease (PD). Normal and PD-linked α-synucleins promote toxicity by relocating to the nucleus where they bind to histones and inhibit their acetylation. Administration of histone deacetylase (HDAC) inhibitors restores the histone acetylation state and rescues α-synuclein-mediated toxicity (Kontopoulos et al., 2006). Remarkably, administration of HDAC inhibitors to ALS mouse models successfully decreases cell toxicity (Yoo and Ko, 2011).

### Implications for ALS pathogenesis

Loss or depletion of VAPB activity in zebrafish, worms and mice induce a relatively mild phenotype possibly because of a functional redundancy between closely related VAP proteins in these models (Kabashi et al., 2013; Han et al., 2012). In *Drosophila*, only one *VAP* gene is present and therefore its inactivation leads to the appearance of a more aggressive mutant phenotype (Pennetta et al., 2002; Forrest et al., 2013). Mice expressing the VAPB-P56S transgene have been generated and they exhibit no overt neurodegenerative phenotype (Aliaga et al., 2013; Qiu et al., 2013; Tudor et al., 2010; Kuijpers et al., 2013b). However, a recent report describes degeneration of a subset of motor neurons in mice expressing higher levels of the VAPB-P56S transgene (Aliaga et al., 2013). In addition, neurodegeneration has been associated with the expression of the same transgene in cell culture systems (Langou et al., 2010; Suzuki and Matsuoka, 2011; Teuling et al., 2007). Taken together these considerations further underscore the importance of VAPB dosage in inducing ALS-like phenotypes, including neurodegeneration. It is not uncommon that genetic models fail to reproduce the full range of clinical manifestations associated with a human disease and for this non-human primate models should represent a better option. Genetic models, however, remain invaluable tools to dissect mechanistic aspects of disease pathogenesis.

Studies in several model systems showed that the P56S mutation induces ALS by a loss-of-function mechanism, possibly due to a dominant negative effect (Ratnaparkhi et al., 2008; Tsuda et al., 2008; Suzuki et al., 2009; Forrest et al., 2013; Kuijpers et al., 2013a). Furthermore, a reduction in VAP protein levels have been reported in sporadic ALS patients, SOD1 mutant mice as well as in induced pluripotent stem cells derived from ALS patients (Teuling et al., 2007; Anagnostou et al., 2010; Mitne-Neto et al., 2011). The dominant negative effect of P56S mutation is further supported by the observation that the mutant protein accumulates into intracellular inclusions, which also sequester the wild-type protein (Teuling et al., 2007; Chen et al., 2010). Here we uncover a novel mechanism for the disease pathogenesis and we show that increasing the levels and/or activity of DVAP may be detrimental as well. The notion that increased hVAPB levels are sufficient to induce neurodegeneration would be greatly supported by finding that duplications or mutations in the non-coding regions of the *hVAPB* gene are associated with ALS in humans. For *hVAPB*, however, such mutations have yet to be identified. These and other studies indicate that, in many cases, a strict dichotomy between loss-of-function versus gain-of-function mechanisms do not respond to a biological reality as both mechanisms seem to be at play (Robberecht and Philips, 2013). In support of this hypothesis, variations in the dosage of the SMN gene have been shown to induce two closely related motor neuron diseases as loss-of-function mutations in SMN cause Spinal Muscular Atrophy while duplications of the same gene have been linked to ALS (Bürglen et al., 1995; Blauw et al., 2012).

Finally, our data are compatible with a model in which, in DVAP-V260I-induced ALS, gain- and loss-of-function mechanisms may coexist within the same neuron depending on the subcellular compartment. At the presynaptic compartment, a local increase in DVAP levels induces a remodeling of synaptic morphology and microtubule architecture compatible with a gain-of-function mechanism. Conversely, in neuronal cell bodies the inherent ability of wild-type hVAPB to be included into aggregates that may also function as a sink for other important proteins, suggests that DVAP could be pathogenic by a possible loss-of-function mechanism. The complexity of such a scenario pinpoints to novel opportunities for possible therapies but at the same time makes the task of finding an effective therapeutic strategy particularly challenging.

## Materials and Methods

### Fly strains and husbandry

Flies were reared in standard cornmeal–molasses medium and maintained at room temperature unless otherwise specified. To drive the expression of DVAP transgenes in neurons, larval muscles and adult eyes we used the *elav-Gal4* line (Lin and Goodman, 1994), the *BG57-Gal4* line (Budnik et al., 1996) and the *ey-Gal4* line (Halder et al., 1998; Hauck et al., 1999), respectively. We used the *ey-Gal4* instead of the *GMR-Gal4* driver because *GMR-GAL4* has been shown to induce eye neurodegeneration on its own (Rezával et al., 2007). Transgenic expression in adult muscles was achieved by using the *MHC-Gal4* line.

### Generation of DVAP-WT and DVAP-V260I transgenic lines

Site-directed mutagenesis on DVAP cDNA was performed using the Quick Change Site Directed Mutagenesis Kit (Agilent, Colorado Springs, CO, USA) following manufacturer's instructions. Gal4-resposive transgenic lines were constructed cloning DVAP-WT and DVAP-V260I cDNAs in the pUAST vector (Brand and Perrimon, 1993) using the cloning strategy previously described (Pennetta et al., 2002). Constructs were injected into embryonic germ cells and transgenic lines were established following standard procedures (Genetic Services, Inc., Sudbury, MA, USA). Given the strong dosage-dependent effect on mutant phenotypes observed for both the *DVAP-WT* and *V260I* transgenes, in generating the transgenic lines we opted for the method of random transgene insertion instead of that in which transgenes are inserted at specific sites (Bischof et al., 2007). Using this approach, we generated 8 independent transgenic lines expressing different levels of either DVAP-WT or DVAP-V260I proteins.

### Genetics

For temperature pulses, to induce a higher expression of the Gal4 protein, flies of the relevant genotypes were left to mate at room temperature for 20–24 hours and then the parents were removed while the progeny was shifted to 30°C in a water bath. Only for the *ey-GAL4* experiments, crosses and rearing of animals were performed at 25°C because at higher temperatures adult flies fail to eclose, possibly due to an ectopic, toxic expression of the transgene in areas of the nervous system other than the eye.

### Immunohistochemistry

To study NMJ morphology, wandering third instar larvae were dissected and processed as previously described (Pennetta et al., 2002). Control genotypes used for analysis were F1 larvae from Canton-S males crossed to the homozygous females of the relevant GAL4 driver line. Primary antibodies are rabbit anti-HRP antibodies (1:500, Jackson ImmunoResearch Laboratories, West Grove, PA, USA), mouse anti-Futsch 22C10 monoclonal antibody (1:200, Developmental Studies Hybridoma Bank, Iowa City, IA, USA), rabbit anti-Lamin antibody (1: 500), guinea pig anti-DVAP antibody (1:500, Pennetta et al., 2002) and mouse anti-Hsp70 (1:200, Affinity Bioreagents, Rockford, IL, USA). Secondary antibodies were used at a 1:200 dilution.

### Confocal analysis and imaging

Confocal z-stacks were acquired on a Nikon A1R (Nikon, Kingston upon Thames, Surrey, UK) using an ×60/1.4 NA oil immersion plan-apochromat with a sampling rate of 0.4×0.4×0.27 µm in *x*, *y* and *z*, respectively. For multicolour images a channel series protocol was used to minimize bleed through. The same confocal gain settings were applied to control and transgenic samples. Images were processed using Imaris v 7.3.0 (Bitplane, Zurich, Switzerland). For z-stacks, iso-surfaces were created in surpass mode for both lamin and TO-PRO3; whole nuclei were selected from the 3D image volume and measurements for volume, and object bounding box size were acquired. Imaris measurement-pro was used to measure the distances between nuclei along each muscle fibre. To measure the circularity of nuclei within muscles, images were analyzed with ImageJ software (ImageJ software, National Institute of Health, Bethesda, MD, USA). Circularity is defined as 4π×Area/Perimeter^2^, where 1 represents a perfect circle and 0 an infinitely elongated polygon. For visualization of DVAP within the nucleus a 3D-volume view of DVAP and TO-PRO3 was combined with an iso-surface of the lamin and a contour plane was used to cut into the lamin volume and show the localization of DVAP within the nucleus.

### NMJ morphological analysis

To quantify the overgrowth phenotypes at the NMJ, body wall muscle preparations were labeled with the presynaptic marker anti-HRP and two different parameters were measured. The total number of synaptic boutons was counted at muscles 12 and 13 of abdominal segment A3 and the percentage of satellite boutons (satellite×100/total boutons) on muscles 6 and 7 of the abdominal segment A3 was also determined. Satellite boutons at the NMJs were defined, identified and quantified according to Torroja et al. (Torroja et al., 1999). The number of loop containing boutons was counted on muscles 4 of the abdominal segments A2 and A3 and expressed as a percentage of the total number of boutons for each NMJ.

### Quantification of the distance between myonuclei

A nearest-neighbor analysis was performed to quantify the distance between nuclei along a given myofibre. A nearest-neighbor analysis determines the average distance between a nucleus and its single closest neighbor within a muscle. To identify the closest neighbor for each nucleus within a muscle, the distance between the center of a nucleus and the center of each closely surrounding nucleus was measured. Only the shortest distance between a nucleus and its nearest neighbor was recorded per each nucleus within a muscle and the average of these distances was taken as a measure of the shortest distance between nuclei in a given muscle.

### Statistical analysis

Statistical analysis including graphing was performed using GraphPad 5.0 (GraphPad Software Inc, LaJolla, CA, USA). For all the experiments, a one-way ANOVA test was applied to the samples. Tukey's multiple comparison tests were used as a post-hoc test when a significant difference was found in the ANOVA test. Data throughout the paper are presented as mean ± s.e.m. A minimum of 10 larvae were analyzed per every genotype. At least 20 flies per genotype were analyzed in scoring the adult eye phenotype.

### Analysis and quantification of the *Drosophila* adult eye phenotype

For the eye morphology, two-dimensional images were obtained using a Nikon D5100 DSLR camera attached to a SZX9 Nikon stereomicroscope (Nikon, Kingston upon Thames, Surrey, UK). To quantify the eye surface area images were analyzed with ImageJ software. For histology, flies were raised at 25°C and collected immediately after eclosion. Heads were severed and place in fresh fixative (ethanol:formaldehyde:acetic acid at 85:10:5) overnight at 4°C. Heads were then washed and placed in 70% ethanol and processed into paraffin using standard histological procedures. 10 µm serial sections were obtained and rehydrated with PBS. Sections were stained with H&E.

### Adult phenotypes

Eclosion rates were determined by counting numbers of empty versus full (dead) pupae on the sides of vials in which flies of different genotypes had been allowed to lay for comparable time periods. Food was covered with a thick layer of yeast powder to avoid that eclosed flies with an aberrant wing posture would get stuck to the food. Flies exhibiting an aberrant posture in their wings were collected and their phenotype was recorded using an Olympus ZEX stereomicroscope (Olympus, Hamburg, Germany) equipped with a Nikon D5100 DSLR camera.

## Supplementary Material

Supplementary Material
