# Supplementary material for: Gain-of-function mutations in the ALS8 causative gene VAPB have detrimental effects on neurons and muscles
Source: Biol Open. 2013 Dec 10;3(1):59–71. doi: 10.1242/bio.20137070 (PMC3892161; doi:10.1242/bio.20137070)
Supplement: Supplementary Material [file supp_3_1_59__index.html]

Gain-of-function mutations in the ALS8 causative gene VAPB have detrimental effects on neurons and muscles — Gain-of-function mutations in the ALS8 causative gene VAPB have detrimental effects on neurons and muscles — Supplementary Material 

# Gain-of-function mutations in the ALS8 causative gene VAPB have detrimental effects on neurons and muscles

## bio.20137070 Supplementary Material

**Files in this Data Supplement:**

- Supplementary Material - Mario Sanhueza et al. doi: 10.1242/bio.20137070
