# Supplementary material for: Gain-of-function mutations in the ALS8 causative gene VAPB have detrimental effects on neurons and muscles
Source: Biol Open. 2013 Dec 10;3(1):59–71. doi: 10.1242/bio.20137070 (PMC3892161; doi:10.1242/bio.20137070)
Supplement: Supplementary Material [file supp_bio.20137070_bio.20137070-s1.pdf]

Biology Open

Supplementary Material

Mario Sanhueza et al. doi: 10.1242/bio.20137070

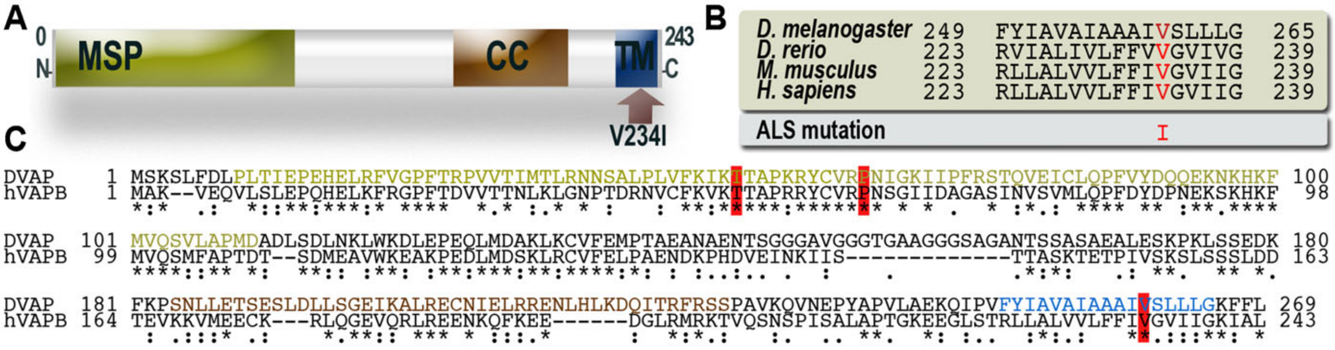

Fig. S1. Identification of the V234I/V260I mutation on VAP proteins. (A) Predicted functional domains in hVAPB: a transmembrane domain (blue) at the C-terminus, a coil-coiled domain (brown) in the middle and a domain (green) at the N-terminus showing a significant homology to the nematode major sperm protein (MSP). The Val234 residue (indicated by an arrow) is located within the transmembrane domain and it has been replaced by an Isoleucine. (B) High degree of conservation of the transmembrane domain in VAP proteins from different species. (C) Sequences of human VAPB (hVAPB) protein and its *Drosophila* orthologue DVAP were aligned by using the ClustalW version 1.82 alignment program available from EMBL–EBI. Asterisk indicates an identity match; colon indicates conservation between amino acids with strongly similar properties (scoring >0.5 in the Gonnet PAM 250 matrix) and period indicates a conserved substitution between amino acids with weakly similar properties (≤0.5 in the Gonnet PAM 250 matrix). Red boxes highlight the amino acid residues changed by the ALS8 causing mutations identified so far. The *Drosophila* protein (DVAP) exhibits the same functional domains as the hVAPB.

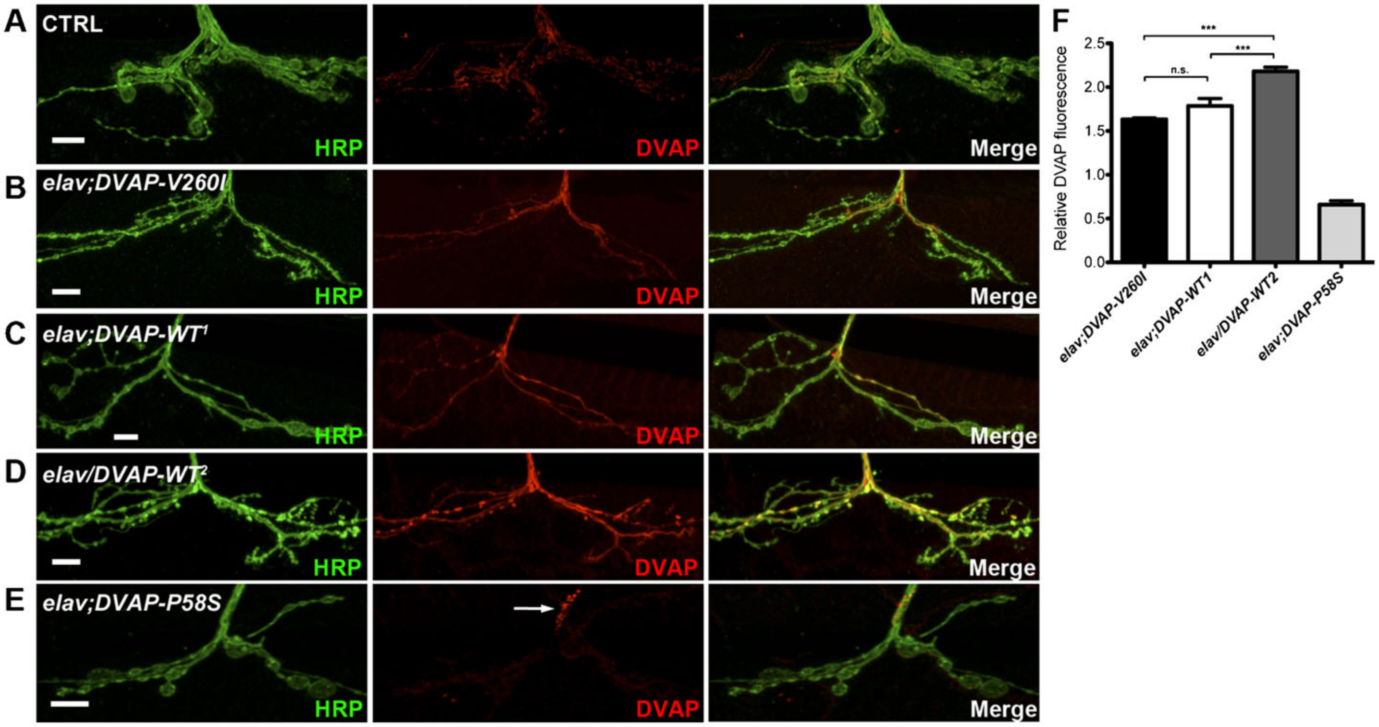

Fig. S2. Synaptic levels of DVAP in different genetic contexts. (A) Control *elav/+* NMJs and (B) *elav;DVAP-V260I*, (C) *elav;DVAP-WT*^{1}, (D) *elav/DVAP-WT*^{2}, (E) *elav;DVAP-P58S* NMJs stained with antibodies specific for DVAP and for anti-HRP. Scale bars: 10 μm. (F) Quantification of synaptic DVAP intensity for the reported genotypes. DVAP fluorescence intensity is presented as a relative ratio of fluorescence intensity values of DVAP against those of controls. Note that in DVAP-P58S transgenic line aggregates are evident in the terminal part of the nerve (arrow in panel E) and the endogenous protein at the synapse has decreased to about 66±4% of the control value. Conversely, compared to controls DVAP levels at the synapse are significantly increased in all the other transgenic lines ($P<0.001$ in all cases). However, upregulation of DVAP levels in neurons expressing *DVAP-V260I* is comparable to that induced by the *DVAP-WT*^{1} transgene ($P>0.05$) and significantly lower than that associated with the strongest *DVAP-WT* overexpressing line DVAP-WT^{2} ($P<0.001$). The difference in DVAP levels between DVAP-WT^{1} and DVAP-WT^{2} lines was statistically significant ($P<0.001$).

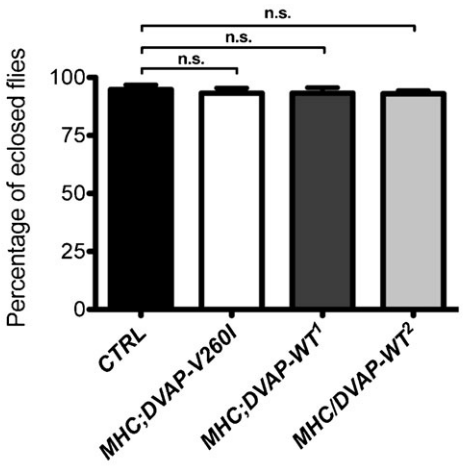

Fig. S3. Overexpression of *DVAP-WT* and transgenic expression of *DVAP-V260I* in muscles does not affect viability. Eclosion rate of flies of the indicated genotypes. *MHC-Gal4/+* flies were used as controls. P>0.05.

Biology Open
